# Safe by Design Flow Technology for Diazoacetonitrile Generation and Direct Conversion to Pyrazoles

**DOI:** 10.1002/open.202600006

**Published:** 2026-04-09

**Authors:** Dušan Bošković, Stefan Loebbecke, Calogero Giancarlo Piscopo, Ligia Johanna Radulescu, Maud Schwarzer

**Affiliations:** ^1^ Energetic Materials Department, Fraunhofer Institute for Chemical Technology ICT Pfinztal Germany

**Keywords:** continuous flow, diazoacetonitrile, green methods, hazardous chemistry, pyrazole

## Abstract

Diazo compounds enable short and efficient synthetic routes but their broader adoption is limited by hazards associated with their preparation, storage, and handling. Here, a safe by design continuous flow process is presented in which diazoacetonitrile (DAN) is generated in aqueous solution, transferred into an organic phase with minimal hold up, and immediately consumed in a telescoped [3 + 2] cycloaddition with activated alkynes to obtain 3 cyano 1H pyrazoles. Operating windows for solvent choice, temperature, residence time, and pressure are established, and the influence of added HCl on nitrosation efficiency and DAN decay is observed. Dichloromethane and benzonitrile are compared as extraction and reaction media: benzonitrile enables higher organic phase DAN concentrations, whereas dichloromethane facilitates isolation. Under optimized conditions, NMR yields of ∼60%–80% are obtained for reactions with methyl propiolate, and a 2 h telescoped run affords a 37% isolated yield after recrystallization. The workflow prevents isolation or accumulation of DAN, improving the safety of processes involving this energetic intermediate and illustrating how continuous flow and biphasic extraction can support greener synthesis by avoiding reaction steps.

## Graphical abstract

A safe by design flow process generates diazoacetonitrile (DAN) in water, transfers it with minimal hold up into an organic phase, and immediately consumes it in a telescoped [3 + 2] cycloaddition. Mapping of solvent, temperature, pressure, and residence time windows enables controlled DAN handling and practical access to cyanopyrazoles.

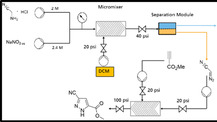

## 1 Introduction

The rapid development of continuous flow technology, particularly in micro and millireactors, has renewed interest in highly reactive intermediates that were historically avoided due to safety concerns [1, 2, 3, 4, 5]. Such systems offer precise control over mixing, heat transfer, and residence time, reducing the instantaneous inventory of hazardous species and enabling safer access to transformations that are difficult to manage in batch. Diazo compounds are a prime example. They provide exceptionally efficient routes to high value molecules through fast, selective transformations [5, 6, 7, 8]. Yet their wider adoption remains limited by the risks associated with their preparation, storage, and handling [6, 7, 8, 9]. Among them, diazoacetonitrile (DAN) is particularly noteworthy: while less explored than ethyl diazoacetate (EDA), it offers unique reactivity paired with significant safety challenges. DAN was first prepared by Curtius in 1898 via nitrosation of aminoacetonitrile in batch [10]. Subsequent attempts to reproduce or scale the process were repeatedly complicated by violent decomposition events, underscoring that even modest accumulation of DAN in unstirred zones or warm spots can trigger explosive behavior [10]. This sensitivity made DAN notorious and discouraged its use for decades. A safer batch protocol was later developed by Pettit and Dewar using diethyl ether as a diluting medium [11], but the inherent risk arising from stored or isolated DAN remained a central limitation. Despite these challenges, DAN possesses several attractive features. Its enhanced stability relative to diazomethane—arising from conjugation between the diazo and nitrile groups—allows it to act as both nucleophile and electrophile [8, 9]. This dual reactivity opens concise synthetic routes not easily accessible with other 1,3 dipoles. In recent years, Mykhailiuk and Koenigs demonstrated that DAN can significantly improve the safety and sustainability profiles of various transformations when generated and consumed in controlled environments [12, 13, 14, 15]. Such approaches reduce global warming potential (GWP) and human toxicity potential (HTP) by shortening reaction sequences and minimizing auxiliary reagents and waste [16, 17, 18, 19]. In 2015, Mykhailiuk introduced an acetonitrile based in situ generation of DAN [13], followed by the continuous aqueous synthesis reported by Empel et al [14]., marking important steps toward safer handling. Beyond these advances in production, DAN serves as a reliable 1,3 dipole for [3 + 2] cycloadditions with electron deficient alkynes to yield cyanopyrazoles [12, 13, 20, 21, 22, 23, 24]. These heterocycles are often difficult to prepare through conventional multistep routes, which require several demanding functional group transformations and typically involve reagents or conditions that are far less sustainable than a single convergent cycloaddition, therefore providing a beneficial exemplary application for DAN. The value of this transformation is illustrated by compounds such as Darolutamide, a clinically relevant cyanopyrazole derivative [25, 26]. Building on the progress in aqueous, in situ DAN generation, we present here a safe by design continuous flow process that forms DAN in water, immediately transfers it into an organic phase, and consumes it without isolation. The approach minimizes hazardous DAN inventory through controlled nitrosation, fast biphasic mass transfer, and immediate downstream conversion. Operational windows for temperature, pressure, solvent, and residence time are mapped to balance nitrosation rate, DAN stability, and extraction efficiency. This telescoped process provides a practical and inherently safer way to exploit DAN in synthesis while aligning with strategies broadly applied in fine chemical and pharmaceutical manufacturing [27, 28].

## 2 Results and Discussion

### 2.1 Standard Setup and Preliminary Tests

Recent studies have shown that synthesizing DAN (**3**) and simultaneously extracting it from the aqueous phase provides an efficient route for continuous processing [5, 15].

**FIGURE 1:**
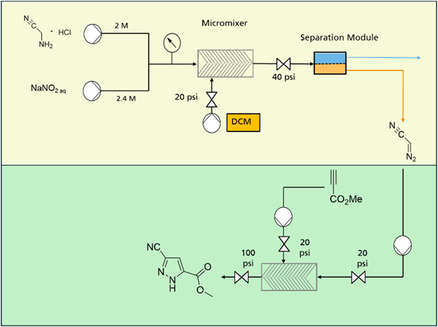
Experimental setup used for the telescoped synthesis of cyanopyrazoles, comprising DAN synthesis (yellow, upper section) and [3 + 2] cycloaddition (green, lower section).

**SCHEME 1:**
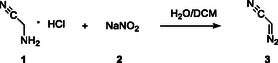
DAN (**3**) synthesis from aminoacetonitrile hydrochloride (**1**) and sodium nitrite (**2**).

To enable a telescoped synthesis, the first step optimized was the continuous production and extraction of DAN, as outlined in Scheme 1. Experiments used the setup in Figure 1, an improved version of the previously reported system [5]. All IR ATR measurements reported here were performed offline.

Aqueous solutions of aminoacetonitrile hydrochloride (**1**) and sodium nitrite (**2**), together with dichloromethane, were pumped into a glass microreactor. Pressure was controlled by a backpressure regulator. Downstream of the backpressure valve, the reaction stream passed through a membrane module for phase separation. Technical details of the setup and materials are provided in the Supporting Information.

**FIGURE 2:**
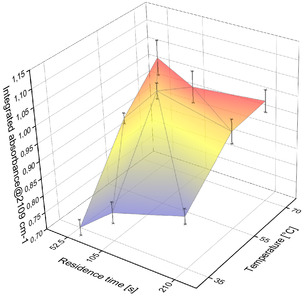
DAN (3) synthesis: Effect of residence time and temperature.

Optimization of the continuous DAN synthesis (Figure 2) as guided by earlier results [5], which identified 70°C and 2.8 bar as optimal for this setup.

### 2.2 Study of the Effect of Hydrochloric Acid

**FIGURE 3:**
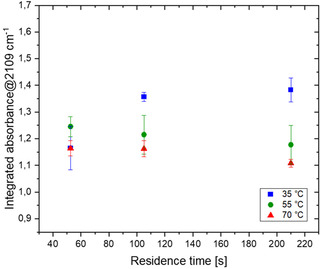
DAN (3) synthesis in the presence of 0.4 M HCl (1:5 molar ratio to aminoacetonitrile hydrochloride).

The first parameter that was investigated, seeking further improvement in the reaction performance, was the effect of hydrochloric acid in the reaction mixture (Figure 3). The presence of mineral acid is indeed expected to accelerate the formation of the DAN by promoting the formation of nitrosating species (e.g., NO^+^, N_2_O_3_) from sodium nitrite under acidic conditions [8, 9].

Added mineral acid was evaluated to modulate nitrosation. Using aminoacetonitrile hydrochloride introduces acid; additional HCl was therefore tested to match or approach nitrite stoichiometry. At 35°C, added HCl increased organic phase DAN concentrations across residence times. Though, it clearly appeared that HCl has a no pronounced effect on DAN production at higher temperatures. Indeed, while the formation of nitrosonium ion is promoted, this effect is likely overbalanced by a faster DAN decomposition.

**FIGURE 4:**
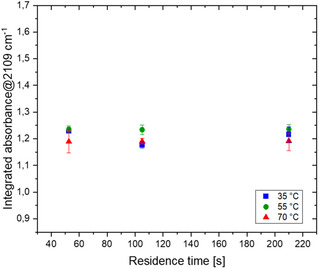
DAN (3) synthesis in the presence of 0.2 M HCl (1:10 molar ratio to aminoacetonitrile hydrochloride).

A subsequent series of experiments was carried out reducing the HCl concentration to 0.2 mol/L, to obtain more accurate information under milder reaction conditions (Figure 4).

In this case, the results of the experiments performed at 35°C are more difficult to rationalize, most probably because of concurrent decomposition and reaction rate modification induced by the HCl without a clear prevalence of any of those.

A general improvement of the DAN production rate has been observed at 55°C. However, this effect is not proportional to the residence time; prolonged reaction time is associated with more DAN decomposition. Running the reaction at 70°C with a residence time of 52 s results in almost identical DAN formation, independently from the addition of HCl, proving that the catalytic effect is compensated by the decomposition of DAN. Already in the presence of 0.2 M HCl at 70°C, the decomposition of DAN entirely jeopardizes the positive effect of longer residence time.

Besides, due to increased gas evolution, the results registered at 55°C and, particularly at 70°C, are affected by a larger error.

### 2.3 Investigation of Different Solvent Systems

**FIGURE 5:**
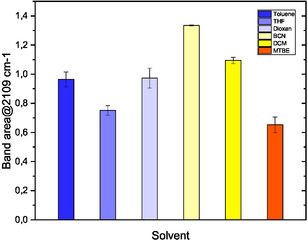
DAN (3) synthesis: Comparison of different extraction solvents at 55°C.

Previous reports on the preparation of DAN employed chloroform or dichloromethane as extraction solvents [5, 12, 13, 15], both of which raise environmental and regulatory concerns for potential scale up. In a safe by design flow process, however, the primary constraint is the need to limit DAN accumulation, and any alternative solvent must support rapid extraction and immediate downstream consumption of DAN under controlled conditions. For this reason, the evaluation of candidate solvents was performed at one well defined operating point (55°C, 105 s residence time), which represents a robust and safe regime for highlighting the practical advantages and drawbacks of different media. Five organic solvents were therefore screened as potential alternatives to chlorinated solvents (Figure 5) to determine which solvents could sustain stable biphasic extraction and reaction performance without compromising the safety driven design of the process. While improved environmental profiles are desirable, the results confirm that the ability to maintain low DAN inventory and reliable flow operation remains the decisive criterion for solvent selection in this context. Dioxane was immediately discarded, although it delivered satisfactory results in terms of DAN production rate, this solvent is miscible in water, withdrawing the positive effect of developed DAN synthetic process. The other ethers tested, methyl‐*t*‐butylether (MTBE) and tetrahydrofurane (THF) displayed a much lower DAN extraction rate compared to DCM. Toluene gave moderate DAN concentrations, lower than DCM. Instead, the use of benzonitrile (BCN) resulted in a 20% increase in the DAN extraction rate compared to DCM, therefore this aromatic solvent was selected for further optimization tests.

**FIGURE 6:**
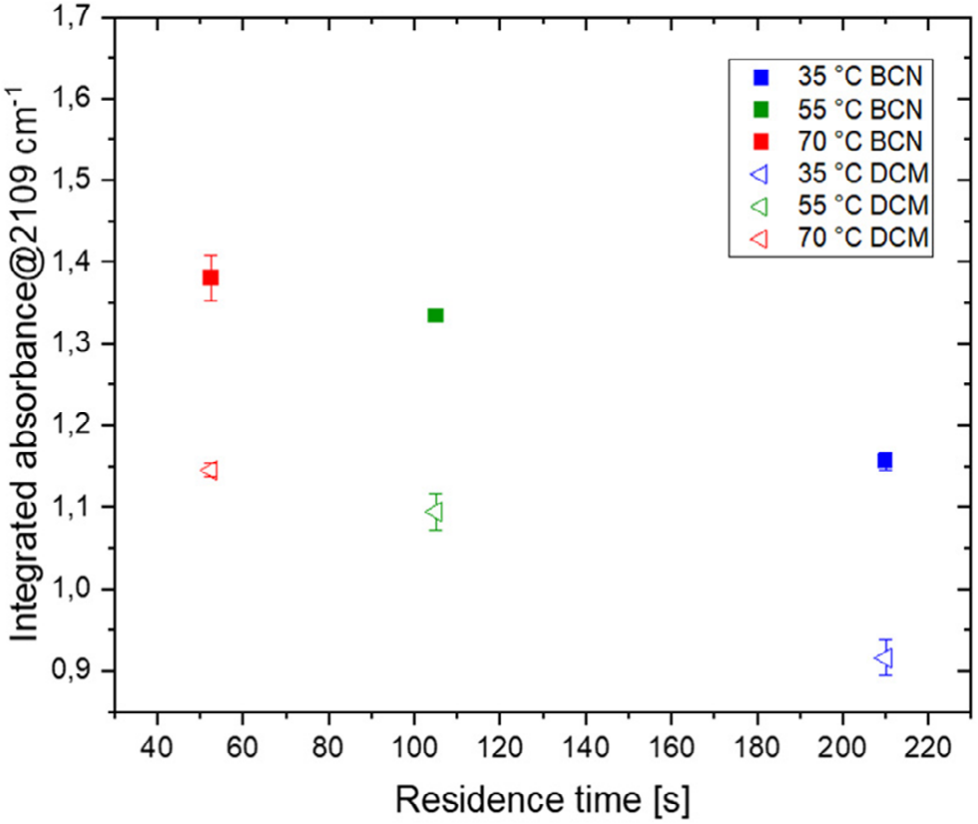
DAN (3) synthesis: Comparison of H_2_O/DCM and H_2_O/BCN solvent systems.

Based on the results of Figure 5, it was chosen to perform the reaction in BCN at the best residence time/temperature combination, to rapidly assess the influence of the solvent system under different reaction conditions. Results reported in Figure 6 show the same pattern for both solvent systems, confirming the higher extraction efficiency observed for BCN also at different temperatures and residence times. Under these conditions, the best results were obtained at 70°C with a residence time of 52 s.

**FIGURE 7:**
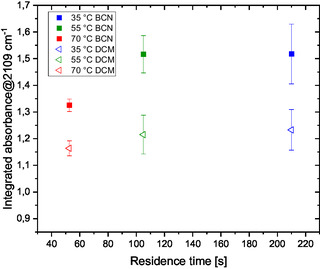
DAN (3) synthesis: Comparison of H_2_O/DCM and H_2_O/BCN solvent systems in the presence of 0.4 M HCl.

The following experiments were carried out to test the use of BCN as extraction solvent in the presence of HCl (Figure 7). The results follow the same trend as for DCM: the best performance occurs at 35°C with a residence time of 210 s. Nearly identical results are achieved at 55°C with a 105 s residence time, which provides the highest productivity. It can be assumed that BCN promotes faster extraction, reducing the DAN decomposition in the water phase.

### 2.4 [3 + 2] Cycloaddition of DAN with Methylpropiolate

**SCHEME 2:**
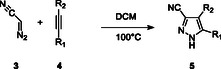
[3 + 2] Cycloaddition of DAN (**3**) with alkynes (**4**).

After the identification of the best reaction conditions for the continuous preparation of DAN, the telescoped synthesis of cyano pyrazoles was the following target of this experimental work. DAN can readily react with alkynes bearing electron‐withdrawing moieties through a [3 + 2] cycloaddition giving the cyanopyrazole according to Scheme 2 (details about the alkynes are reported in Table 1).

**TABLE 1:** Alkynes 4 employed in the [3 + 2] cyclization with DAN 3.

4	R_1_	R_2_
a	–COOMe	H
b	–COOMe	–COOMe
c	–COOEt	–COOEt

The continuous synthesis of cyanopyrazoles was performed with an experimental setup consisting of two syringe pumps, a glass micromixer, a capillary reactor, and three back pressure valves.

The telescoped [3 + 2] cycloaddition with methyl propiolate **4a** was investigated by varying residence time, temperature, flow rate, and reactor volume, using a large stoichiometric excess of DAN. Conversion was quantified by the disappearance of the alkyne sp H (1H‐NMR, δ ≈ 2.99) and formation of an aromatic sp^2^ H in the pyrazole ring (δ ≈ 7.17). Several experiments were conducted varying residence time, temperature and reactor volume. Changing the reactor volume, without consistent differences in the residence time, allows to perform the reaction at different flow rates, obtaining important indications on the relevance of the mass transport in the reaction.

The results are summarized in Table 2.

**TABLE 2:** Continuous flow synthesis of cyanopyrazole 5a through [3 + 2] cyclization.

Entry	Temperature, °C	Residence time	Pressure, bar	Yield, %
1^a^	70	3 min 5 s	8,4	5
2^a^	70	5 min 9 s	≈8.5	6
3^a^	70	7 min 43 s	≈8.5	8
4^b^	50	2 min 27 s	≈8.5	3
5^b^	50	4 min 56 s	8,5	5
6^b^	70	8 min 11 s	8,2	13
7^b^	70	4 min 56 s	8,5	10
8^b^	100	4 min 56 s	8,5	26
9^b^	100	8 min 11 s	8,2	33
10^b^	100	12 min 16 s	8.0	42
11^c^	70	9 min 27 s	9.0	13
12^c^	70	15 min 45 s	8,6	17
13^c^	70	23 min 38 s	8,5	34
14^c^	100	23 min 38 s	8,2–10	57
15^c^	80	15 min 45 s	8,3–10	36
16^c^	80	23 min 38 s	6,0 ‐10	41
17^c^	90	23 min 38 s	7,0 ‐ 13	59
18^c^	90	15 min 45 s	6,2	40
19^c^	100	9 min 27 s	10,0–12	36
20^c^	100	15 min 45 s	10,0–12	60
21^d^	90	22 min 15 s	10,0–12	63
22^e^	90	22 min 15 s	10,0–12	64

a Glass reactor + PTFE capillary of 3.4 mL volume.

b glass reactor + 8 m 1/16″ capillary tube of 5.4 mL volume.

c glass reactor + 8 m PTFE coil 1/16″ capillary tube + 4.5 m additional plastic coil 1/8″ with total volume of 10.4 mL.

d 8 m plastic coil 1/16″ capillary tube + 4.5 m additional PTFE coil 1/8″ with total volume of 8.9 mL.

e same setup as d double amount of alkyne 4a.

The reported results clearly highlight an improvement in the yield of the synthesis of cyanopyrazole **5a** proportional to the temperature and residence time. Significant results were obtained only by reaction temperature higher than 70° and residence time of at least 5 min. The best yield was registered running the reaction at a temperature of 90°C and a residence time of 22 min (entry 21), reaching 63% of cyanopyrazole **5a**. At higher temperatures, reactor pressure increased, due to clogging with a black solid, so those conditions were avoided.

Similar good results were obtained at the temperature of 100°C and a residence time of almost 16 min (entry 20).

Comparing attempts carried out at the same temperature using PTFE capillary of different lengths, it was difficult to point out a clear trend, due to the differences in residence time (the flow rate can only be set according to digital values). In general, it seems that better mixing and therefore better reaction outcomes can be achieved with bigger reactor volumes and consequent higher flow rate, as appears from entries 3 and 6.

Entry 17 shows that the use of a glass mixer microreactor doesn’t introduce any advantage to the reaction, quite the opposite a slight decrease in the yield to 59% has been observed. It appears evident that by such a prolonged reaction time the more efficient mixing at the beginning does not affect the mass transport within the reactor. In this attempt the residence time was slightly increased using the same flow rate compared to entry 21, due to the bigger reaction volume. The yield is, however, consistently higher than the one reported for the experiment of entry 18. Higher temperatures and longer residence times lead to clogging of the reaction capillary due to the formation of a black solid. It can be assumed this substance is generated from the decomposition and polymerization of DAN.An additional experiment was carried out with double amount of the alkyne **4a** (entry 22), no significative increase in yield (64%) for the cyanopyrazole **5a** has been registered.

### 2.5 Investigation of Different Alkynes and Solvents

The [3 + 2] cyclization of DAN was tested with two additional alkynes to prove a broader applicability of the developed method (Table 3).

**TABLE 3:** Continuous flow synthesis of cyanopyrazole 5 through [3 + 2] cyclization.

Entry	Temperature, °C	Residence time	Alkyne	Yield, %
1	90	23 min 38 s	a	59
2	100	15 min 45 s	a	60
3	90	23 min 38 s	b	62
4	100	15 min 45 s	b	80
5	90	23 min 38 s	c	—
6	100	15 min 45 s	c	—

Using the alkyne **4b,** an improvement in the yield of cyanopyrazole has been observed, reaching a yield of 80% for the product **5b**. The electron‐withdrawing effect of two –COOMe groups has hence a positive effect on the cycloaddition reaction.

Conversely, employing the alkyne **4c** bearing two –COOEt groups, a complex mixture of products has been formed, preventing the quantification of the desired product **5c**. In fact, although the electron poor alkynes are activated toward [3 + 2] cycloadditions are also more sensitive to competing side reactions, which likely reduced the selectivity.

Further experiments were performed aiming to find a green alternative to DCM as solvent, based on the results obtained for the synthesis of DAN it was tested whether benzonitrile represented a valid option. Due to abundant formation of oligomers and clogging problems during preliminary tests at 100°C the reaction temperature was set to either 80°C or 90°C. The results on Table 4 are compared to a standard experiment with DCM at 90°C employing glass reactor connected to 8 m capillary tube (1/16″ diameter) plus 4.5 m additional PTFE coil (1/8″ diameter) for a total volume of 10.4 mL (entry 1). Using BCN the yield increases steadily from 80°C to 90°C at each tested residence time, reaching the maximum value of 59% at 90°C and with a residence time of almost 24 min (entry 6). The results are therefore almost identical to the ones achieved with DCM. Another experiment was carried out using *p*‐tolunitrile (TCN) as solvent (in this case without glass mixer); this resulted in a drastic decrease of the yield to 13% (entry 7).

**TABLE 4:** Continuous flow synthesis of cyanopyrazole **5a** through [3 + 2] cyclization: solvent investigation.

Entry	Temperature, °C	Residence time	Solvent	Yield, %
1	90	23 min 38 s	DCM	59
2	80	9 min 27 s	BCN	38
3	90	9 min 27 s	BCN	47
4	80	15 min 45 s	BCN	43
5	90	15 min 45 s	BCN	52
6	90	23 min 38 s	BCN	59
7	90	22 min 15 s	TCN	13

Benzonitrile appears as possible choice for the [3 + 2] cycloaddition of DAN, however using the DCM it is possible to further improve the yield pushing the temperature to 100°C. Besides, the isolation of the product is much simpler from a volatile solvent such as DCM compared to BCN.

Based on these findings, it was set to perform one telescoped reaction, starting from aminoacetonitrile **1** to obtain the cyanopyrazole **5a** in DCM as proof of concept.

The reaction was performed under the optimized reaction parameters for both steps, namely 70°C and 105 s residence time for the synthesis of DAN **3**, followed by the cyclization with methyl propiolate **4a** at 90°C and a residence time of 22 min and 15 s. The continuous reaction was operated under the reported conditions for 2 h allowing the cyanopyrazole 5a after recrystallization in a 37% yield. This value is considered acceptable, owing to the fact that the isolation process is quite complicated and especially recrystallization is associated with a consistent loss of product.

## 3 Conclusion

A safe by design continuous flow protocol was developed for the generation of DAN in water, its rapid transfer into an organic phase, and its immediate consumption in a telescoped [3 + 2] cycloaddition to access 3 cyano 1H pyrazoles. The process establishes operational windows for temperature, residence time, pressure, and solvent, and clarifies the influence of mineral acid on the balance between nitrosation efficiency and DAN decay. Under optimized conditions, methyl propiolate affords 60%–80% NMR yields, while a 37% isolated yield is achieved after recrystallization in a 2 h telescoped experiment—reasonable given the gap typically observed between NMR yield and isolated material when mild and safe purification conditions are required. Both dichloromethane and benzonitrile serve as effective extraction and reaction media with clear process trade offs: dichloromethane supports higher temperature operation and simplifies isolation, whereas benzonitrile offers similar reaction performance at 80°C–90°C but complicates product recovery due to its high boiling point. The biphasic configuration minimizes DAN decomposition and enhances inherent safety while maintaining practical productivity. Improved performance at increased flow rates suggests enhanced mass transfer, while limitations include DAN instability at extended residence times or elevated temperatures and the complexities of isolating product from high boiling solvents. Future work expanding substrate scope and deepening mechanistic insight into DAN formation and decay under biphasic flow is expected to support further improvements, including opportunities for automation and digital process control to strengthen safety and reproducibility.

## Supporting Information

Additional supporting information can be found online in the Supporting Information section.

## Funding

This work was supported by the Fraunhofer‐Gesellschaft (Lighthouse project ShaPID).

## Conflicts of Interest

The authors declare no conflicts of interest.

## Supporting information

Supplementary Material

## Data Availability

The data that support the findings of this study are available from the corresponding author upon reasonable request.
