# Supplementary material for: Safe by Design Flow Technology for Diazoacetonitrile Generation and Direct Conversion to Pyrazoles
Source: ChemistryOpen. 2026 Apr 9;15(4):e202600006. doi: 10.1002/open.202600006 (PMC13063248; doi:10.1002/open.202600006)
Supplement: Supplementary file 1 — Supplementary Material [file OPEN-15-e202600006-s001.pdf]

Supporting Information

Safe-by-Design Flow Technology for Diazoacetonitrile Generation and Direct Conversion to Pyrazoles

Dr. Dušan Bošković, Dr. Stefan Loebbecke, Dr. Calogero Giancarlo Piscopo, Ligia Johanna Radulescu, Maud Schwarzer

Experimental

Materials and methods

Chemicals: Aminoacetonitrile hydrochloride (AAN-HCl 98+% Thermo Scientific), sodium nitrite (p.S. Sigma Aldrich), dichloromethane (DCM); Cyclisation: methyl propiolate (99% Alfa Aesar), dimethyl sulfone (*Trace*CERT® by Sigma-Aldrich).

Equipment: pumps: HiTec Zang SyrDos, Sykam, Harvard PHD 2000, bath: IKA, reactor LTF, Upchurch pressure valve, membrane separation module Zaiput SEP-10.

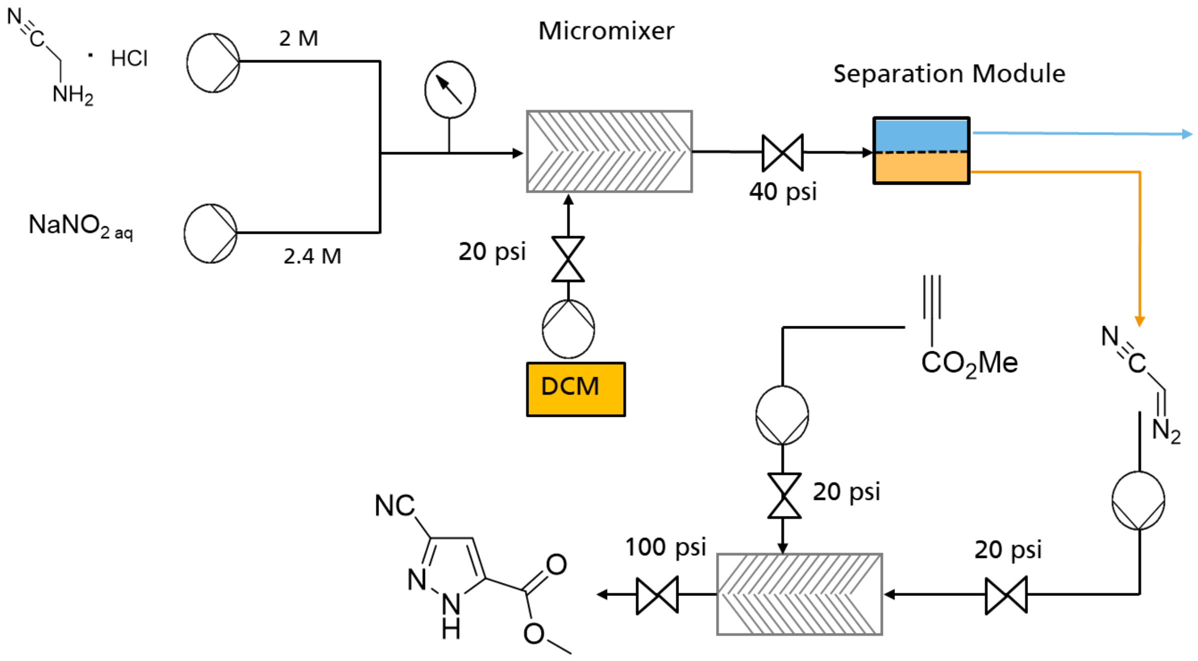

Figure 1. Setup for the telescoped synthesis of cyanopyrazoles via DAN.

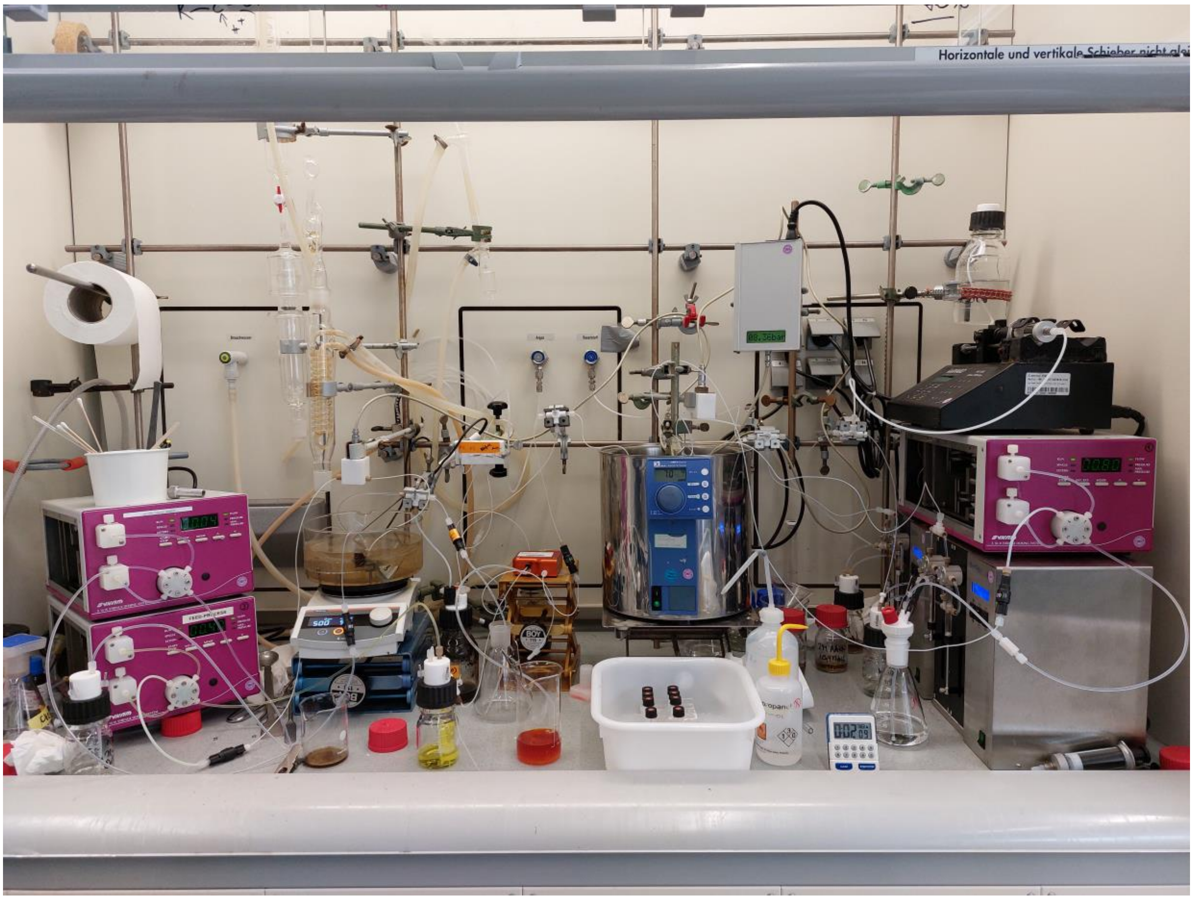

*Figure 2. Photo of combined setup for DAN **3** synthesis (right) and cyclisation (left)*

Synthetic procedures
Experimental procedure

Formation of DAN. 2 M aqueous AAN (flow rate 0.20 mL/min), 2.4 M aqueous sodium nitrite (flow rate 0.20 mL/min) and dichloromethane (flow rate 0.40 mL/min) were delivered by pumps via 1/16" PTFE capillaries into a glass microreactor in a water bath. The reactor consists of a single channel with a chaotic mixing structure (ST design, Little Things Factory) with the following approx. dimensions: a hydraulic diameter of 0.5 mm, a channel length of 86 cm and a volume of 1.4 mL. A back-pressure regulator maintained the pressure at 40 psi. Downstream of the valve, the two-phase stream was separated using a Zaiput SEP-10 membrane module. Samples of each phase were collected and cooled in an ice bath for prompt ATR IR measurement.

Cyclisation. Typical setup and procedure: The DAN solution was dried over Na_2SO_4. This solution and a second solution of methyl propiolate (20 vol% in DCM) were pumped into a glass microreactor (same design as above) followed by a PTFE coil (8 m of 1/16" i.d. and 2.5 m of 1/8" i.d.; combined volume 10.4 mL) immersed in an oil bath at 90°C. Flowrates were 0.60 mL/min of DAN solution and 0.06 mL/min of methyl propiolate solution. After a three-way valve for rapid pressure relief, a back-pressure regulator maintained the pressure at 100 psi. Samples were collected and prepared for NMR-spectroscopy.

Given residence times are calculated from the nominal flow rates and the known reactor volumes. This approach ensures repeatability and is standard practice, as the actual residence time cannot be measured directly in this system due to gas evolution during nitrosation, which causes fluctuations in linear velocity.

Reported pressures are the mean readings from an inline sensor placed upstream of the BPR. Spring-based BPRs regulate to a nominal opening pressure (specified in psi), and gas formation leads to oscillations around this setpoint. These conventions provide consistent, reproducible operational parameters suitable for replication with commercial pumps and standard BPRs

Analytical methods

Off-line ATR-IR spectra of the aqueous and DCM phases were recorded on a Thermo Scientific Nicolet iS50 FT-IR equipped with a diamond ATR plate and a flow cell for sample introduction. Parameters: 32 scans for background and sample; three measurements per sample; three samples per experiment. Spectra were collected from 4000 cm^{-1} to 400 cm^{-1} with a DTGS detector. The flow cell improved repeatability for the DCM phase (volatility) and the aqueous phase (gas formation). Spectra were integrated from 2125 to 2070 cm−1 (asymmetric diazo stretch) using Bruker OPUS and Spectragryph. Although the diamond ATR crystal absorbs in this region, the band intensity enabled quantitative measurements.

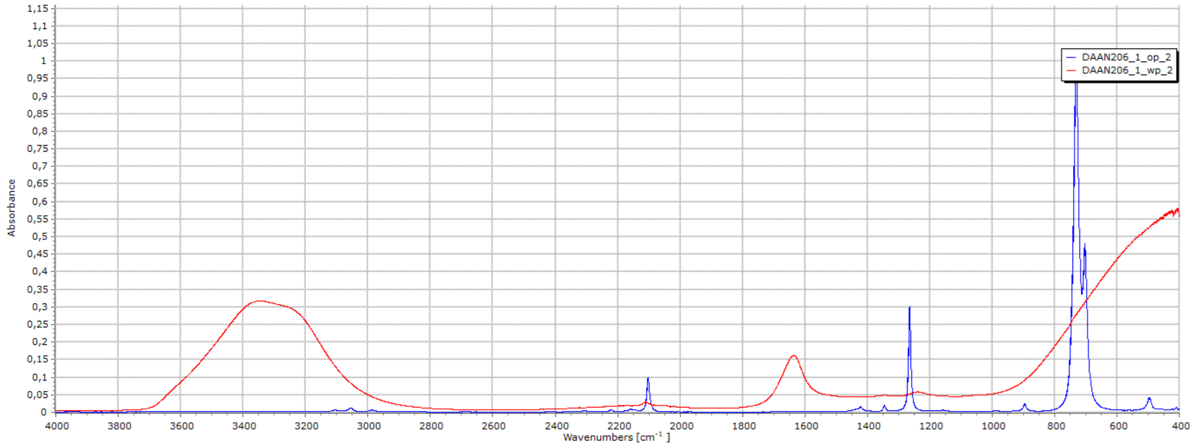

Figure 3. Example of IR spectra of organic phase (blue) and water phase (red) of one sample

NMR: For the cyclisation of DAN and methyl propiolate measurements were taken with a Nanalysis 60 MHz benchtop NMR spectrometer. Parameters: 64 scans, spectral width 12 ppm, 8192 points. The reaction solution was applied directly; the dichloromethane ^{1}H signal was used as lock. We compared the signal intensities of the C-H signals of the methyl propiolate reactant (2.99 ppm) and product (7.17 ppm) to calculate percentage and of CH_3 (reactant 3.76, product 3.93) for consistency.

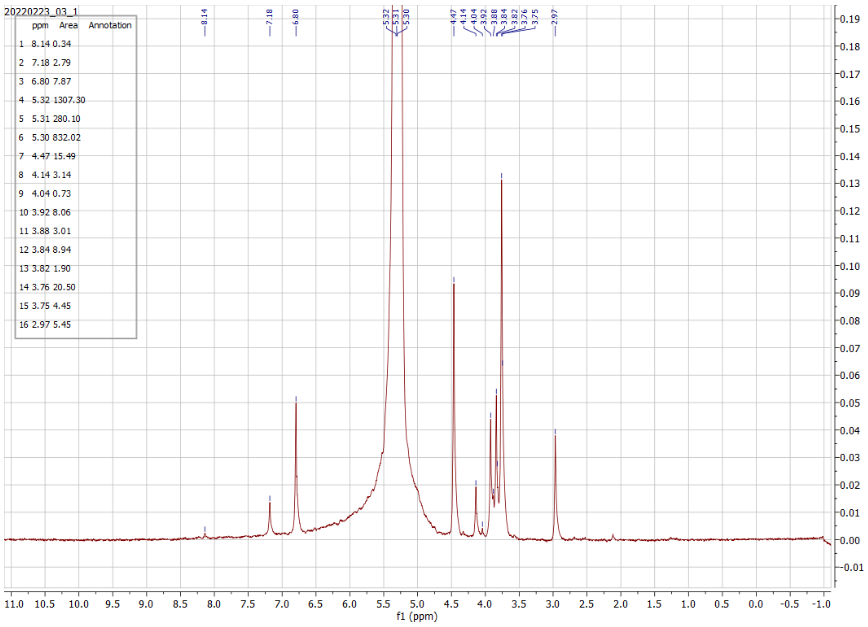

*Figure 4. Typical 1H-NMR spectrum of the cyclisation product **5** solution.*
